# Morphological Changes in the Bone Marrow of the Dogs with Visceral Leishmaniasis

**DOI:** 10.1155/2014/150582

**Published:** 2014-03-05

**Authors:** Claudia Momo, Ana Paula Prudente Jacintho, Pamela Rodrigues Reina Moreira, Danísio Prado Munari, Gisele Fabrino Machado, Rosemeri de Oliveira Vasconcelos

**Affiliations:** ^1^The Postgraduate Program on Veterinary Medicine, FCAV-UNESP, Jaboticabal, SP, Brazil; ^2^Departamento de Ciências Exatas, FCAV-UNESP, Jaboticabal, SP, Brazil; ^3^Departamento de Clínica, Cirurgia e Reprodução Animal, FMVA-UNESP, Araçatuba, SP, Brazil; ^4^Departamento de Patologia Veterinária, Faculdade de Ciências Agrárias e Veterinárias (FCAV-UNESP), Via de Acesso Professor Paulo Donato Castellane, s/n°, Bairro Industrial, 14.884-900 Jaboticabal, SP, Brazil

## Abstract

The aim of this study was to evaluate the most frequent lesions in the bone marrow of dogs naturally infected by *Leishmania (Leishmania) chagasi.* Thirty-three dogs sacrificed at the Zoonosis Control Center of Araçatuba, a municipality endemic for visceral leishmaniasis (VL), were used. The animals were classified as asymptomatic, oligosymptomatic, and symptomatic groups. At the necropsy, bone marrow samples were collected from the femur, fixed, processed, and stained with hematoxylin and eosin. The lesion intensity was classified as mild, moderate, or severe. The parasite load was determined using immunohistochemistry. The most important lesions consisted of multifocal to diffuse granulomas, megakaryocytic dysplasia, and medullary aplasia. There were no statistical differences between the three clinical groups regarding parasite load and lesion intensity. Asymptomatic dogs also presented high parasitism in the bone marrow as dogs with clinical signs of VL. It was concluded that, regardless of clinical group, the bone marrow is a site for multiplication of *Leishmania chagasi*. Possibly, the bone marrow dysplasia may arise from the presence of many parasitized and activated macrophages in this organ. Consequently, it affects the profile of hematopoietic cells in the bone marrow and systemic circulation.

## 1. Introduction

Visceral leishmaniasis (VL) is a zoonosis with worldwide distribution, caused by protozoa *Leishmania*. The members of its parasitic evolutionary cycle include domestic and wild mammals, humans, and vector insects. In the Americas, the etiological agent involved is *Leishmania (Leishmania) chagasi. *This protozoon is an obligate intracellular parasite of cells of the mononuclear phagocytic system of vertebrate hosts. In Brazil, the vectors of *Leishmania chagasi *are the blood-sucking phlebotomines *Lutzomyia longipalpis *and *Lutzomyia cruzi *[1]. 

The protozoon shows a preference for multiplying in the host's lymphoid organs. Bone marrow and popliteal lymph nodes are the main organs used in parasite investigations, with the aim of diagnosing canine VL in endemic areas [1]. Among the many clinical manifestations, infected dogs may present prolonged coagulation times, epistaxis, hematuria, and other signs of hemorrhagic diathesis, caused by vasculitis, uremia, liver failure, platelet sequestration by the spleen, and thrombocytopenia due to medullary aplasia [2].

The bone marrow is usually densely parasitized in infected dogs. Initially, the erythropoiesis and granulopoiesis are normal, but functional imbalance occurs in these processes, during the more advanced phases of the infection, and leads to diminished cell production, with repercussions on the hematological condition. This change occurs by histiocytic hyperplasia, erythrocytic hypoplasia and, after evolution, for aplasia [3].

Myelodysplastic syndromes are acquired changes that occur in hematopoietic stem cells in dogs, cats, and humans. These disorders can be primary or secondary and are characterized by anemia and/or leukopenia and thrombocytopenia, with one or several dysplastic changes in the bone marrow [4]. In humans, myelodysplastic syndromes are found in cases of VL. The observed pancytopenia is correlated with splenomegaly, hemolysis, inefficient erythropoiesis, reticuloendothelial hyperplasia with abnormal iron retention by macrophages, and autoimmune hemolytic anemia [5].

Studies on the different pathological aspects of the organs affected by *Leishmania chagasi *infection are essential. Some studies on lymph nodes and the spleen have already been conducted, but little attention has been given to histopathological changes to the bone marrow. Therefore, the objectives of the present study were to analyze the main lesions of the bone marrow in dogs naturally infected with *Leishmania chagasi* and compare these findings with the parasite load and clinical staging of these dogs.

## 2. Material and Methods

The dogs used in this study came from the Zoonosis Control Center of Araçatuba (SP, Brazil), where they were put down because of a positive diagnosis of canine VL, in accordance with the serological methods recommended by the Ministry of Health in Brazil (enzyme-linked immunosorbent assay—ELISA test). Thirty-three dogs were used, without preference for age, breed, or sex. They were put down under barbiturate anesthesia (Thiopental, Cristália Itapira, SP), as recommended in the code of ethics for use of animals in scientific research [6], by means of an intravenous injection of potassium chloride. A necropsy was conducted on the dogs immediately after death. In an external examination on the cadavers, the macroscopic changes were assessed, thereby enabling classification in the groups of this study [1]: nine symptomatic dogs (group S), which presented skin lesions, onychogryphosis, and cachexia, among other clinical signs; 16 oligosymptomatic dogs (group O), with lymphadenomegaly and slight weight loss; and eight asymptomatic dogs (group A), without apparent clinical signs. Before necropsy, all dogs were subjected to cytological analysis of bone marrow and popliteal lymph node to confirm the presence of the parasite.

Bone marrow samples were removed from the femur for subsequent histological and immunohistochemical analysis. These were fixed in a 10% formol solution and buffered with phosphates (pH 7.2) for 12 hours. They were then processed and embedded in paraffin, and sections of 5 *μ*m in thickness were then cut and stained with hematoxylin and eosin. The intensity of the inflammatory reaction and the predominant cell type were determined by means of scores, such that the cell response was classified as mild (1), moderate (2), or severe (3).

The parasitic load from the bone marrow was evaluated by immunohistochemical technique, and serum from a positive dog for VL was used as primary antibody (modified from Tafuri—[7]) at a dilution of 1 : 1000. The bone marrow sections were deparaffinized and hydrated. Antigen recovery was done by means of steam (Philips Walita steaming pan), with a 10 mM sodium citrate solution (pH 6.0), for 40 minutes. Endogenous peroxidase activity was blocked through incubation in a solution of methyl alcohol and hydrogen peroxide 3% (30 volumes), in a darkened chamber at room temperature for 10 minutes. To block nonspecific proteins, a commercial product was used (Protein Block, DakoCytomation, code X0909), at room temperature for 20 minutes. Between each of the steps described above, the material was immersed in baths of distilled water and in phosphate-buffered solution (PBS), at pH 7.2, for five minutes. The primary antibody (dog hyperimmune serum) was incubated in a damp chamber at 4°C, at a dilution of 1 : 1000, for 18 hours. The biotinylated secondary antibody and the substrate of streptavidin peroxidase (LSAB kit, DakoCytomation, code K0690) were incubated at room temperature in a damp chamber for 30 minutes. The reaction was developed using the chromogen diaminobenzidine (DAB, DakoCytomation, code K3468) and was counterstained using Harris hematoxylin. The negative control for labeling amastigote forms of *Leishmania *sp. consisted of running the procedure with PBS (pH 7.2), instead of the primary antibody.

To determine the percentage of immunolabeled cells, five microscope fields were examined (with 40x objective lens). In this manner, the mean number of immunolabeled cells per animal was obtained, for each group of infected dogs. Statistical analysis was done using the nonparametric Kruskal-Wallis test and Dunn's multiple comparison test, with comparisons between the groups of dogs. The Graphpad Prism statistical software (version 4.00, 2003) was used for all the analyses, and differences were taken to be significant when *P* < 0.05.

## 3. Results and Discussion

In the macroscopic analysis on the dogs, in addition to the lymphadenomegaly lesions, onychogryphosis, and skin disorders, among others, there were also clinical signs of anemia characterized by pallid mucosa, runnier and watery dark red blood, and bone marrow of pallid to whitish coloring, especially in the dogs in group S. Most of the dogs in all three clinical groups presented levels of infestation with ectoparasites (fleas and ticks), which may have contributed towards their anemic condition. Nonetheless, the possibility of immunomediated anemia cannot be dismissed, given that hypergammaglobulinemia is an important characteristic in dogs infected with VL and that anti-erythrocyte antibodies have been described in the literature as a cause of anemia in humans. Other factors may also be involved, such as increased permeability of the erythrocyte membrane and cytokines that interfere in the action of erythropoietin and sequestration of erythrocytes to the spleen [8]. The hemosiderosis observed in macrophages that were present in the bone marrow was identified in 37.5% of the dogs in the group A, 25% of the animals in the group O, and 33.3% of the dos in group S (Table 1). This abnormality coincided with the macroscopic finding of pallid mucosa and bone marrow. The presence of hemosiderosis was possibly related to conditions of immunomediated hemolytic anemia. In human patients with VL, iron retention by macrophages has been described, associated with conditions of anemia [5]. In studies that evaluated hemograms from dogs with VL, it was found that there were reductions in the concentrations of red blood cells, hemoglobin, and hematocrit and in the numbers of lymphocytes and monocytes in animals with high densities of parasites in the bone marrow [9].

**Figure 1:**
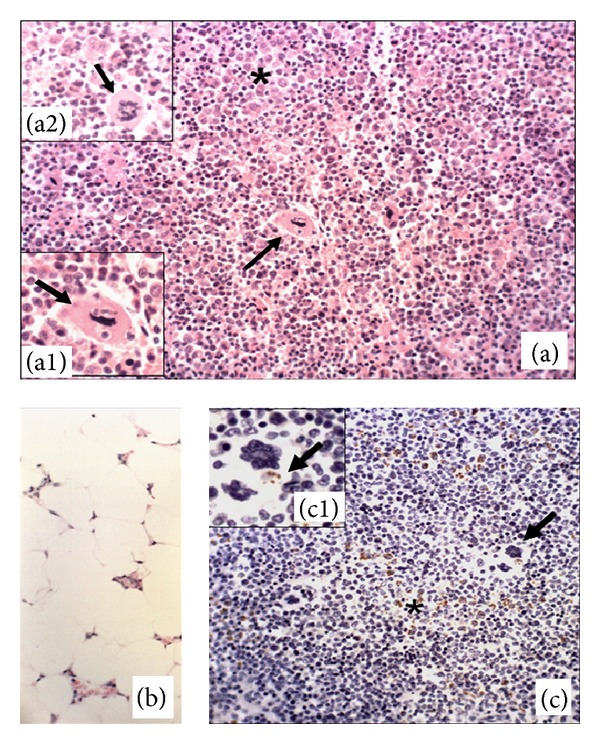
Photomicrographs of the bone marrow of dogs with visceral leishmaniasis. (a) Granuloma (∗), megakaryocytic emperipolesis (arrow, (a1), group O), and dysplastic megakaryocytes ((a2), group O). (b) Bone marrow with severe aplasia (group A); hematoxylin and eosin, objective 40x. (c) Immunolabeling of amastigote forms of *L. chagasi *in macrophages (∗); detail of megakaryocytic emperipolesis with an immunolabeled amastigote form (arrow, (c1); symptomatic dog); streptavidin-biotin-peroxidase complex, objective 40x.

Analysis on bone marrow samples under a microscope showed that the most frequent lesions were granulomas (Figures 1(a) and 1(c)), in association or not with reductions in the population of hematopoietic cells (Figure 1(b)), in 31.25% of the oligosymptomatic dogs (5/16), 37.5% of the asymptomatic (3/8), and 44.4% of the asymptomatic (4/9). The macrophages that formed these granulomas presented a very variable parasite load (Figure 1(c)). The granulomas were diffuse, without typical nodule formation, in situations with large quantities of intracytoplasmic parasites. In addition to the macrophages, the presence of plasmocytes and, to a lesser degree, well-differentiated lymphocytes was noted. These results were similar to the other studies that described that macrophages (granulomas) and plasmocytes were the main inflammatory cells present in lymph nodes in cases of lymphadenopathy in dogs with VL [10, 11].

Analysis on the hematopoietic cells of this lymphoid organ showed that the megakaryocytes presented emperipolesis (Figure 1(a1)) and dysplasia (Figure 1(a2)). The megakaryocytic dysplasia was characterized by fragmentation of the nucleus or abnormal lobulation. Megakaryopoiesis is regulated by substances that stimulate cell differentiation (interleukin 1, IL-3, IL-6, IL-11, IL-13, and erythropoietin) or that inhibit it, such as platelet factor IV and transforming growth factor *β* (TGF-*β*) [12]. In symptomatic dogs, a profile consisting predominantly of Th2 cytokines has been described, such as IL-10 and TGF-*β* in the spleen and liver [13] and in peripheral lymph nodes [14]. Future studies are needed to verify whether there is an abnormal cytokine profile in bone marrow that can cause megakaryocytic dysplasia.

Emperipolesis was observed in seven dogs of the three groups studied, and this abnormality was most frequently seen in the oligosymptomatic group. This process consists of active penetration of one cell into another cell, which remains intact. It differs from phagocytosis, because the penetrating cell can leave the host cell without damage to either of them. Red blood cells, precursors of polymorphonuclear cells, and lymphocytes are among the cells that have been described as capable of this process and which have been observed within megakaryocytes [15]. Emperipolesis has already been described in dogs with VL, and it may occur in myeloproliferative diseases, hemorrhages, and neoplasia in human [4]. It might occur in megakaryocytes stimulated by inflammatory cytokines, in cases of infection with *Leishmania chagasi. *The objective of this intracytoplasmic interaction between two cell types is not clear, but it has been reported that releases of alpha-granular proteins stimulated by cytokines and growth factors produced by megakaryocytes are factors that induce this biological phenomenon [15]. In canine VL, it is possible that certain inflammatory mediators released by activated macrophages may be one of the factors regulating such changes, but further studies are needed to confirm this theory.

It is known that megakaryocytes go through several stages of differentiation before they become platelets. Their ultrastructural phenotype goes on gradually changing during this differentiation. The erythroid transcription factor (GATA-1) has a large influence on this differentiation process. It has been found that mutation of this factor may lead to conditions of thrombocytopenia [12]. In the literature, it can be inferred that an imbalance in the production of cytokines and growth factors are responsible for thrombocytopenia [13, 14]. Future studies are needed to verify whether there is influence of activated macrophages in the canine VL on the megakaryocytic differentiation and if this condition would lead to thrombocytopenia, which have been described in the canine VL [4]. Another hypothesis to be studied would evaluate whether there is an association between thrombocytopenia and damage to the transcription factor GATA-1, which has been incriminated to cause dysplastic megakaryocytes and thrombocytopenia [12].

The reduction in the lymphoid cell population was most evident in one dog in the symptomatic group. On the other hand, some oligosymptomatic dogs presented predominance of lymphocytes and/or precursors of monocytes in the bone marrow. Reduction of T lymphocytes is one of the most important mechanisms used by *Leishmania* sp. to evade the immune system, and this is induced by apoptosis [16]. Moreira [17] studied the lymph nodes of the same dogs as in the present study and found that the density of cells undergoing apoptosis was the greatest in the symptomatic group (*P* < 0.05), thus coinciding with the lymphoid atrophy and with greater parasite load in these lymph nodes. In the bone marrow, this change did not occur specifically in the symptomatic group, and this condition coincided with reductions in all the cell lineages when medullary aplasia was present.

Three dogs in each group presented significant reductions in cell populations (pancytopenia), with predominance of adipose tissue in the bone marrow, thus characterizing medullary aplasia (Figure 1(b)). Most of these dogs were young, that is, between one and three years of age. Pancytopenia has been described in humans with VL [5]. Reduced cellularity in the bone marrow, presence of myelofibrosis, lymphoplasmacytic infiltrate, megakaryocytic dysplasia, and late maturation of erythrocytes and the myeloid lineage were features observed in a cat infected with *Leishmania infantum *and presenting systemic dissemination of the parasite [18]. Some authors reported that, in dogs with VL, dysplasia of the megakaryocytic and erythrocytic lineages correlated with increased production of the cytokines TNF-*α* and IFN-*γ*, which were produced by activated macrophages [4].

Despite reports that polymorphonuclear cells participate in infection caused by *Leishmania chagasi* [19], there was no predominance of this cell population in the bone marrow, except in two dogs in the asymptomatic and oligosymptomatic groups that possibly had some type of secondary infectious process that was recruiting these cells from the bone marrow to other sites, without involvement in VL.

In the immunohistochemical analysis, immunolabeling for the parasite occurred predominantly in the cytoplasm of parasitized macrophages (Figure 1(a)), restricted to the amastigote forms of the protozoon. Out of the 33 dogs studied, 12 did not present immunolabeled parasites in the bone marrow (three in group A, seven in group O, and two in group S), but all these animals were positive on cytological analysis of popliteal lymph node. The differential tropism for organs of the same dog suggests that there is responsiveness to the parasite organ-dependent and related to immune competence of each infected dog.

**Figure 2:**
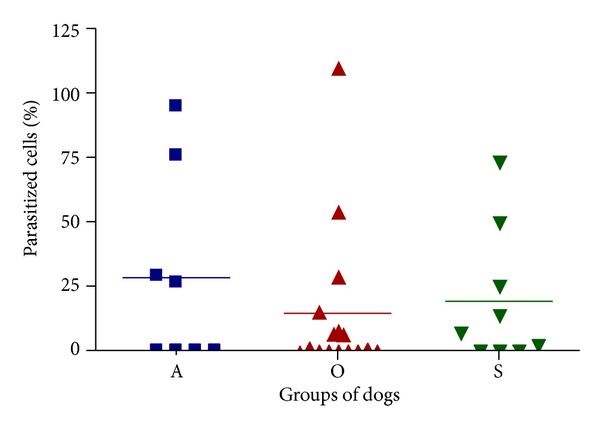
Proportion of parasitized cells in bone marrow of dogs with visceral leishmaniasis. Note the marked variation in mean of infected cells in the three groups of dogs. There was no significant difference between groups by Kruskal-Wallis test (*P* > 0.05).

The positive animals belonged to all three clinical groups, and the highest mean number of parasitized macrophages was 109.8 (in an oligosymptomatic dog). In the asymptomatic dogs, a mean number of 94.8 cells positive for *L. chagasi *was observed. However, no significant differences in parasite load were observed between the three groups of dogs (*P* > 0.05/Figure 2). Other authors have reported increased bone marrow parasite density in infected dogs, predominantly in symptomatic animals [9, 20]. Likewise, higher parasite load was associated with diminished CD4 and CD8 T lymphocyte levels in the peripheral circulation, with lower expression of MHC-II in these cells [9].

In two dogs with high parasite loads, amastigote forms of *Leishmania* sp. were found immunolabeled in the cytoplasm of megakaryocytes (Figure 1(c1)). This suggested that the protozoon was using the phenomenon of emperipolesis. In this study, analysis of the blood count was frustrated due to euthanasia of the animals. In future studies, it would be important to include this parameter in the research, as well as evaluating the profile of circulating leukocytes by flow cytometry to assess the real capacity of the immune response of these animals.

The results from this study allow it to be inferred that invasion of macrophages into the bone marrow (granulomas) may cause damage through changing the architecture of this organ and occupying the space of hematopoietic cells. In the same way, such events may impair the functioning and differentiation of these types of cells because of the release of cytokines, growth factors, and nitric oxide. In other organs, the protozoon *Leishmania *sp. may evade the immune system through inducing apoptosis of T lymphocytes and inhibiting presentation of antigens to T lymphocytes or phagocytosis of the parasite by macrophages [16]. In the bone marrow, it seems that there is interference with cell differentiation and activation of transcription factors, thereby leading to dysplasia of different cell lineages and directly affecting the population of cells present in the peripheral blood. Further studies are needed in order to comprehend the functional changes in the bone marrow in dogs naturally infected with *L. chagasi*.

## 4. Conclusions

In the light of the conditions of this study, the following conclusions could be reached.

There was significant presence of granulomas and high parasite loads in the bone marrow of all three groups of infected dogs.

Myelodysplasia was most important in the megakaryocytes. Bone marrow aplasia was observed in young animals, independent of the clinical group. Bone marrow is an important lymphoid organ in clinical analyses on dogs, since it presents significant changes that explain the hematological condition.

## Figures and Tables

**Table 1:** Proportion of histopathological changes and the mean parasite burden in the bone marrow of dogs with visceral leishmaniasis.

Group	Aplasia	Granuloma	Megakary ocytes*	Hemosiderosis	Plasmacytic infiltrate	Number dogs parasitized	Mean parasite load
A (*n* = 8)	4	3	3	2	4	4	32.3
O (*n* = 16)	3	5	7	3	5	9	11.4
S (*n* = 9)	3	4	4	3	2	6	21.4

Group A: asymptomatic; O: oligosymptomatic; S: symptomatic; *megakaryocytes with either dysplasia or emperipolesis.
